# Retraction: Deficits in the thalamocortical pathway associated with hypersensitivity to pain in patients with frozen shoulder

**DOI:** 10.3389/fneur.2026.1887229

**Published:** 2026-06-01

**Authors:** Frontiers Editorial Office

The publisher retracts the 17 May 2023 article the cited above.

Following publication, the corresponding author contacted the editorial office requesting retraction of this article. The author stated that they unintentionally incorporated neuroimaging datasets from another research group without proper acknowledgment of their contributions through authorship or explicit data-sharing agreements. Unauthorized data use is a breach of Frontiers' guidelines and those of the Committee on Publication Ethics. As such, this article is being retracted.

This retraction was approved by the Chief Executive Editor of Frontiers. The authors received a communication regarding the retraction and had a chance to respond. This communication is recorded by the publisher.

